# Discovery and lead optimisation of a potent, selective and orally bioavailable RARβ agonist for the potential treatment of nerve injury

**DOI:** 10.1016/j.bmcl.2019.02.011

**Published:** 2019-04-15

**Authors:** Maria B. Goncalves, Earl Clarke, Christopher I. Jarvis, S. Barret Kalindjian, Thomas Pitcher, John Grist, Carl Hobbs, Thomas Carlstedt, Julian Jack, Jane T. Brown, Mark Mills, Peter Mumford, Alan D. Borthwick, Jonathan P.T. Corcoran

**Affiliations:** a Neuroscience Drug Discovery Unit, Wolfson Centre for Age-Related Diseases, Guy’s Campus, King’s College, London SE1 1UL, UK; b DrugMolDesign, 15 Temple Grove, London NW11 7UA, UK; c Sygnature Discovery Limited, Biocity, Pennyfoot Street, Nottingham NG1 1GF, UK

**Keywords:** Retinoic acid receptor, Beta agonist, SAR, Neurite outgrowth, Axon regrowth, C286

## Abstract

Oxadiazole replacement of an amide linkage in an RARα agonist template **1**, followed by lead optimisation, has produced a highly potent and selective RARβ agonist 4-(5-(4,7-dimethylbenzofuran-2-yl)-1,2,4-oxadiazol-3-yl)benzoic acid (**10**) with good oral bioavailability in the rat and dog. This molecule increases neurite outgrowth *in vitro* and induces sensory axon regrowth *in vivo* in a rodent model of avulsion and crush injury, and thus has the potential for the treatment of nerve injury.

## Graphical abstract

**Figure:**
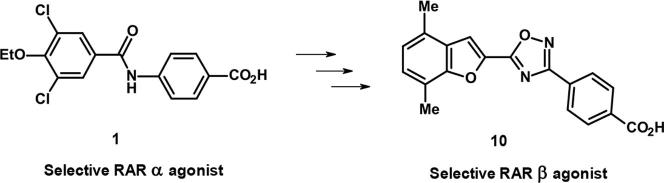

There are no effective treatments for nerve injuries including spinal cord injuries (SCI), stroke, and peripheral nerve injuries. However it has been shown^1^ that stimulating the retinoid signalling pathway in animal models of nerve injury leads to axonal outgrowth and functional recovery. This pathway is activated by retinoic acid (RA) binding to retinoic acid receptors (RAR) that acts in the nucleus to drive the synthesis of RNA and hence produce proteins for axonal outgrowth. Corcoran et al.^2^ have shown that RARβ signalling is required for retinoid mediated neurite outgrowth of neurons. In contrast, signalling by RARα, RARγ or the RXRs has no effect on this action. It has been shown^3^ that the RARβ agonist, CD2019, can activate the RARβ receptor in a dose dependent manner. This initiates axonal outgrowth in models of nerve injury and leads to functional recovery. However CD 2019 is a highly lipophilic compound that is not significantly orally bioavailable and shows only weak to moderate selectivity over RARα and RARγ receptors. AG 261066, more recently described as a selective RARβ agonist is less potent than CD 2019 and less selective than the latter over RARα (Table 4). Our aim was to identify a more drug-like, highly potent and selective RARβ agonist that was orally bioavailable and which had the potential to be useful in the treatment of nerve injury.

Recently, we discovered a novel and selective RARα agonist 4-[(3,5-dichloro-4-ethoxybenzoyl)amino]benzoic acid **1**. This template was the basis of a lead optimisation exercise which led to an orally bioavailable and highly potent RARα agonist with high selectivity against RARβ and RARγ.^4^ As part of this exercise, it was decided to modify the amide linkage between the two rings by replacing it with a variety of 5-membered heterocycles (Table 1). Changing the amide linkage in **1** to thiazole and imidazole gave derivatives **2** and **3** that were weakly active as RARα agonists, but were more potent than amide **1** as RARβ agonists, although only weakly selective for RARβ vs RARα. The oxazole **4** was >40-fold more potent than **1** as an RARβ agonist and had similar agonist potency for all three subtypes.

**Table 1:** Heterocyclic derivatives in RAR α, β and γ transactivation assays.^a^

Compd	Het	∝ EC_50_ nM^a^	β EC_50_ nM^a^	Fold selectivity for β over α^b^	γ EC_50_ nM^a^	Fold selectivity for β over γ^b^	cLogP^d^
**ATRA -**		1.9	**1.2**	**1.56**	0.9	0.75	
**1**	–	46	**1227**	**0.037**	30,000	24	4.4
**2**		240^c^	**120**	**2**	160	1.3	6.1
**3**		594^c^	**423**	**1.4**	ND	–	5.6
**4**		60	**28**	**2.1**	45	1.6	5.5
**5**		18^c^	**1.5**	**12**	28	19	5.1
**6**		31	**110**	**0.28**	5.4	0.05	5.1
**7**		58	**63**	**0.92**	150	2.4	4.3

a Transactivation assays for the RAR alpha, beta and gamma receptors were performed using each of the mouse RAR ligand binding domains. Values usually obtained from three separate experiments. Errors in these assays are approximately 20% of the mean values. Transactivation Assays details see Supplementary data and reference 4. ATRA is all trans retinoic acid.

b The EC_50_ ratios of ∝ to β and γ to β.

c Compound behaves as a partial agonist relative to the amplitude of the normalising ATRA output. All other compounds were determined to be full agonists with their maximum upper asymptote within 20% of that found for ATRA.

d Ref. ^9^.

Surprisingly however, increasing the number of heteroatoms in the heterocyclic ring to give the oxadiazole **5** resulted in a highly potent RARβ agonist and that had 12- and 19-fold selectivity as an agonist over RARα and RARγ respectively. This RARβ agonist selectivity and potency was lost when the isomeric 1,2,4-oxadiazol-5-yl benzoic acid derivative **6** and the 1,3,4-oxadiazol-2-yl benzoic acid compound **7** were examined.

To try and exploit the selective and potent RARβ agonist activity of the 1,2,4-oxadiazol-3-yl benzoic acid derivative **5**, a series of replacements for the 3,5-dichloro-4-ethoxyphenyl ring with other heterocyclic and aryl rings found in known RAR agonists were investigated (Table 2). The 5,5,8,8-tetramethyl-5,6,7,8-tetrahydronaphthalene ring used in AM580,^5^ the 3,5 di-*^t^*butylphenyl ring in Am555,^5^ the 4,7-dimethylbenzofuran ring in ER38925^6^ and the 4-trifluoromethyl-7-fluorobenzofuran ring found in E6060,^7^ were investigated.

**Table 2:** 1,2,4-oxadiazol-3-yl benzoic acid derivatives in RAR α, β and γ transactivation assays.^a^

Compd	X	β EC_50_ nM^a^	α EC_50_ nM^a^	Fold selectivity for β over α^b^	γ EC_50_ nM^a^	Fold selectivity for β over γ^b^	cLogP^d^
**ATRA**	**-**	**1.9**	1.2	**0.9**	0.6	0.5	
**5**		**1.5**	18^c^	**28**	12	19	5.1
**8**		**4200**	18	**17**	0.0043	0.0041	7.2
**9**		**1.4**	4	**3**	2.8	2.1	7.2
**10**		**1.9**	26	**11**	13	5.6	5.3
**11**		**2.5**	19	**5.3**	7.6	2	5.3
**12**		**3.4**	30	**6.3**	9	2	5.8
**13**		**11**	114	**83**	10	7.5	4.1

a–d See Table 1.

Relative to **5**, derivative **8** lost >2700-fold in potency as a RARβ agonist whilst retaining most of its potency at RARα. Compound **9** which retained good RARβ agonist potency, lost all RARβ selectivity and was essentially a potent pan-RAR agonist having a similar potency at all three subtypes. In contrast, the 4,7-dimethylbenzofuran derivative **10** maintained a similar potency and selectivity profile to **5** and as we were keen to move away from the dichlorophenyl motif found in a number of herbicides, this now became our lead compound.

Compared to our lead **10**, the 4-trifluoromethyl-7-fluorobenzofuran **11** and the benzothiophene **12** analogues, are less RARβ/RARα selective while the benzoxazole derivative **13** is less potent as a RARβ agonist (Table 2).

In an attempt to increase further the selectivity and agonist potency of compound **10**, a series of substitutions in the benzoic acid portion of the template were investigated (Table 3). The 2-fluoro compound **14** had a similar level of potency to **10** but lost some RARβ selectivity (Table 3) when compared to **10**. The 2-methyl **15**, 3-fluoro **16** and 3-methyl **17** derivatives all lost considerable potency as RARβ agonists when compared to **10**.

**Table 3:** Derivatives of 4-(5-(4,7-dimethylbenzofuran-2-yl)-1,2,4-oxadiazol-3-yl)benzoic acid in the RAR α, β and γ transactivation assays.^a^

Compd	R	β EC_50_ nM^a^	α EC_50_ nM^a^	Fold selectivity for β over α^b^	γ EC_50_ nM^a^	Fold selectivity for β over γ^b^	cLogP^d^
**10**	**H**	**1.9**	26	**11**	13	5.6	5.3
**14**	**2-F**	**2.2**	16	**8.4**	7.3	3.8	5.1
**15**	**2-Me**	**14**	89	**25**	6.4	1.8	5.5
**16**	**3-F**	**11**	61	**3.7**	5.5	0.33	5.5
**17**	**3-Me**	**47**	600	**14**	13	0.3	5.5

a,b,d See Table 1.

With this information and other data not shown, it became apparent that substitution in the benzoic acid ring in this series did not increase potency at RARβ, which is in contrast to observations made in the analogous RARα agonist series.^4^

The lead RARβ agonist **10** has a high potency at RARβ (similar potency to ATRA) and behaves as a full agonist. It has a selectivity for RARβ over RARα of 13-fold, while selectivity for RARβ over RARγ is 5.6-fold.

Comparison of **10** with the selective RARβ agonist AC-261066^8^ showed that in our hands, **10** is a more potent and selective RARβ agonist (Table 4). Whilst compound **10** is marginally less potent than CD 2019, it has a better selectivity for RARβ over RARα and RARγ and is over two orders of magnitude less lipophilic. The more drug-like template present in **10** translates into a good *in vitro* and *in vivo* profile for this RARβ agonist (Table 5). In comparison to the mouse transactivation data shown in Table 4, we also confirmed that **10** had a similar RARβ potency (EC_50_ = 2.05 nM), similar fold selectivity for RARβ over RARα (23 fold) and for RARβ over RARγ (5 fold) against the human RAR ligand-binding domains,^4^ before further predevelopment studies were investigated.

**Table 4:** Selective RARβ agonists.

Compd	β EC_50_ nM^a^	α EC_50_ nM^a^	γ EC_50_ nM^a^	Fold selectivity for β over α^a^	Fold selectivity for β over γγ^b^	cLogP^d^
**10**	**1.9**	26	11	**13**	5.6	5.3
**AC 261066**	**12**	70	33	**5.8**	2.8	4.9
**CD 2019**	**0.83**	9.2	1.6	**11**	1.9	8.0

a,b,d See Table 1.

The potential drug candidate **10** has excellent physico-chemical properties. It is sufficiently water soluble (>100 µM as the sodium salt) and showed good permeability. The efflux ratios obtained from bi-directional permeability tests was close to unity indicating that **10** is likely not a PGP substrate. With no significant inhibition IC_50_ > 25 μM against five Cyp450 isozymes (1A2, 2C9, 2C19, 2D6, 3A4), a human and mouse plasma protein binding of 98% and 95% respectively and showing very high stability in human microsomes, this compound was progressed to pharmacokinetic studies.

**Table 5:** Physico-chemical and *in vitro* properties of RARβ agonist **10**.

LogD^a^ 7.4	Solubility^b^ µM pH 7.4	MDCK^c^ Papp × 10^−6^ cm/s	MDCK^c^ asymmetry ratio	Cyp450^d^ IC_50_ μM	Human Cl_int_^e^ µL/min/mg protein
2.8	>100	28	0.8	>25	<1

a Measured by shake flask method.

b As the amorphous sodium salt.

c MDR1-MDCK cell line.

d Cyp450 inhibition profile for isoforms 1A2, 2C9, 2C19, 2D6, 3A4.

e Human microsomes incubated with the test compound at 37 °C in the presence of the co-factor, NADPH. The data is the mean on 5 separate experiments. Compound disappearance monitored over 45 min period. SEM is less than 10% of the mean values. For ^a–e^ See Ref. ^9^.

As shown in Table 6 compound **10** was found to possess a promising pharmacokinetic profile in both rat and dog. It demonstrated a low rate of blood clearance, a moderate half-life and good oral bioavailability. It was also found to penetrate the CNS, with nearly equivalent amounts detected in brain tissue when compared to plasma, 8 h after dosing orally to rats.

**Table 6:** Pharmacokinetic data for Compound **10** in Rat and Dog.^12^

Species	Clearance mL/h/kg	Volume distribution ss mL/kg	t_1/2_h	T_max_ h	Fraction absorbed %
rat^a^	3.7	0.41	1.4	1.7	80^b^
dog^c^	1.1	0.23	2.5	1.0	45

a iv dose 0.5 mg/kg administered in 4% DMSO, 38% PEG-400, 58% (0.9%) NaCl. Oral doses of 1, 3 and 10 mg/kg prepared in 8% ethanol and 92% PEG-400.

b Based on mean of data obtained at 1, 3 and 10 mg/kg oral dose levels in comparison to iv dose of 0.5 mg/kg.

c iv dose 0.5 mg/kg administered in 2% DMSO, 98% aqueous hydroxypropyl-β-cyclodextrin (22.5% w/v). Oral dose 3 mg/kg administered in 3% DMSO, 97% aqueous hydroxypropyl-β-cyclodextrin (22.5% w/v). For assay description ^a,c^see Ref. ^4^.

In the HEPG2 cell toxicity assay **10** was found to be completely devoid of alerts at the highest concentration tested (50 μM). Furthermore, in a binding assay for HERG channels, the compound, demonstrated no inhibition at 10 μM. Genetic toxicity testing of the material showed that it was inactive in bacterial cytotoxicity tests up to 100 μM and in an Ames test in three bacterial strains. Similar, negative results were obtained in an *in vitro* micronucleus test in CHO-K1 cells, in both the presence and absence of S9.

Synthesis and characterisation of compounds in Table 1, Table 2, Table 3 have been described^10^ and involve standard preparation of the 5-membered heterocyclic rings. This is illustrated by the preparation of our lead oxadiazole **10** outlined in Scheme 1.

**Scheme 1:**
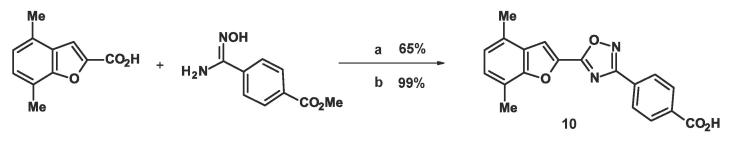
Synthesis of oxadiazole **10**. Reagents and conditions: (a) T3P, EtOAc, DMF, Et_3_N, 0 °C, then warmed to 90 °C and stirred for 18 h; (b) LiOH (2 M, aq.,), THF, 40 °C for 20 h. then at RT HCl (1 M) added (see Supplementary data for full experimental and spectroscopic details).

Compound **10** was evaluated for neurite outgrowth/branching in cerebellar cultures. Cerebellar cultures grown on a monolayer of CHO-MAG were treated with RARβ agonists and neurite outgrowth was assessed by immunostaining and neurite length quantification.^10^ The compound increased neurite length in a dose dependant manner (Fig. 1) and thus has the potential to be useful in the treatment of nerve injury.

**Fig. 1:**
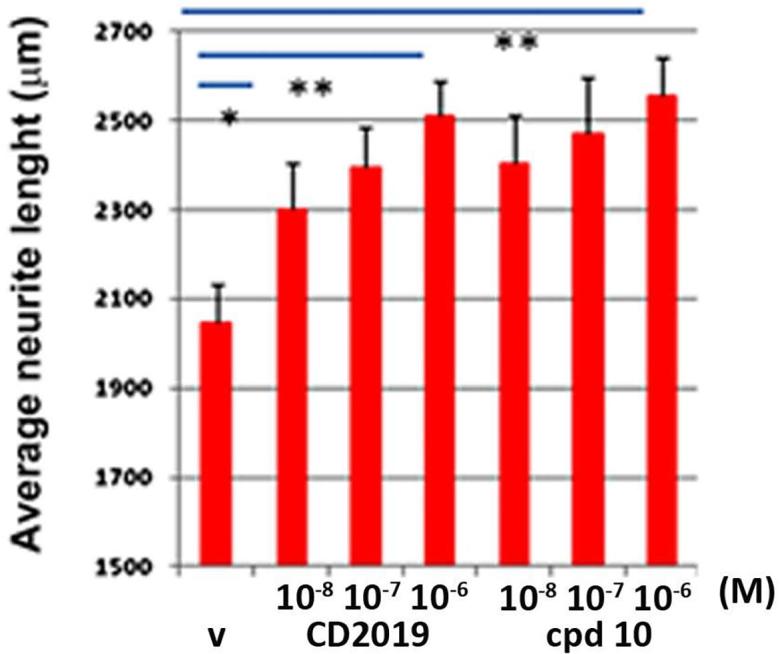
Effects of RARβ agonists **10** on neurite outgrowth. Cerebellar neurons grown on a monolayer of CHO-MAG cells were treated for 24 hr with either vehicle (**V**) or increasing doses of RARβ agonists (1 × 10^−8^–1 × 10^−6^ M). Both RARβ agonists increase neurite outgrowth in a dose dependant manner. Results are means from 3 independent experiments. Statistical analysis was done using Student’s *t*-test between vehicle and each drug’s highest dose. Error bars are SEM and **p ≤ 0.001, *p ≤ 0.01.

The novel RARβ agonist **10** has also been demonstrated to be capable of inducing sensory axon regrowth *in vivo* in a rodent model of avulsion as shown in Fig. 2, where avulsion is defined as the traumatic tear of nerve roots from the spinal cord causing injury. Rats were trained for two weeks prior to surgery in behavioural tasks and scores were recorded the day before surgery, the day after surgery and then weekly, for four weeks. Surgery was performed as previously described.^11^, ^12^ In a sticky tape task, the time taken to sense and remove the tape from the paw of the lesioned forelimbs was measured. From week three of treatment, significantly lower latencies were observed with the injured forelimbs of compound **10** treated rats (3 mg/kg, po) compared to vehicle treated ones. In locomotor tasks, the number of foot slips in a horizontal ladder made by the injured forelimb of the compound **10** treated rats was also measured. This parameter was found to be markedly lower than that of vehicle treated rats from week two. Further details and data on an *in vivo* model of crush injury will be presented in due course.

**Fig. 2:**
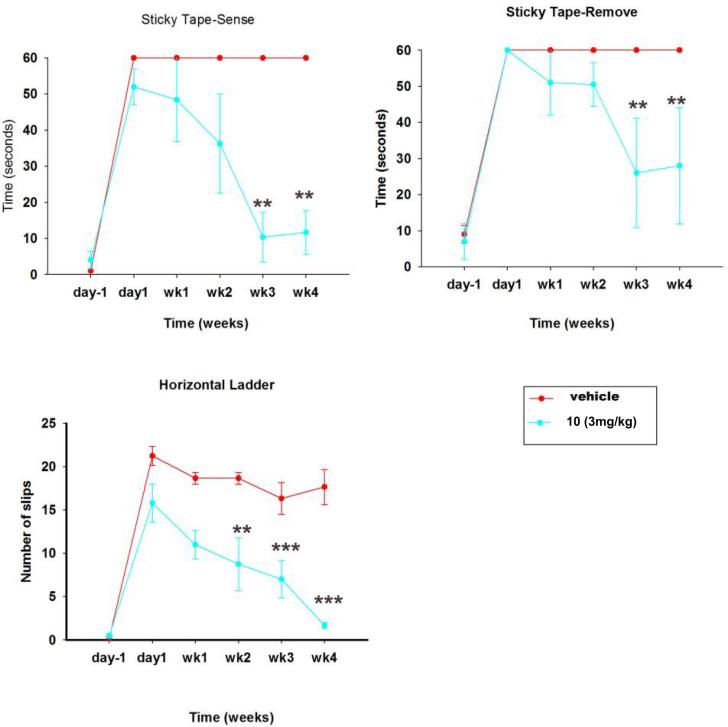
Effects of oral administration of compound **10** in sensory and locomotor functions in avulsed rats. Dose 3 mg/kg, po, three times a week, every other day. Data represent mean ± SEM of *n* = 8, **p ≤ 0.005, ***p ≤ 0.001. Two-way repeated-measures ANOVA, Tukey’s post-hoc test.

In summary, replacing the amide linkage between the two aromatic rings in our selective RARα agonist template with 5-memebered heterocycles, gave compounds which were selective as RARβ agonists. SAR exploration of the oxadiazole based series led to potent and selective RARβ agonists. In particular compound **10**, which will henceforth be referred to as **C286**, possesses favourable physicochemical properties with an oral bioavailability of >40% in both rats and dogs, a good overall PK profile and drug-like properties. Furthermore, it has been shown to be inactive in cytotoxicity and genotoxicity screens. It has also been demonstrated here to increase neurite outgrowth *in vitro* and induce sensory axon regrowth *in vivo* in a rodent model of avulsion and crush injury and thus warrants further consideration as a potential therapeutic agent for the treatment of nerve injury.
