# Corrigendum to “Discovery and lead optimisation of a potent, selective and orally bioavailable RARβ agonist for the potential treatment of nerve injury” [Bioorg. Med. Chem. Lett. 29(8) (2019) 995–1000]

**DOI:** 10.1016/j.bmcl.2019.05.058

**Published:** 2019-08-15

**Authors:** Maria B. Goncalves, Earl Clarke, Christopher I. Jarvis, S. Barret Kalindjian, Thomas Pitcher, John Grist, Carl Hobbs, Thomas Carlstedt, Julian Jack, Jane T. Brown, Mark Mills, Peter Mumford, Alan D. Borthwick, Jonathan P.T. Corcoran

**Affiliations:** a Neuroscience Drug Discovery Unit, Wolfson Centre for Age-Related Diseases, Guy’s Campus, King’s College, London SE1 1UL, UK; b DrugMolDesign, 15 Temple Grove, London NW11 7UA, UK; c Sygnature Discovery Limited, Biocity, Pennyfoot Street, Nottingham NG1 1GF, UK

The authors regret that a mistake was made in the headings of Table 2, Table 3. The correct Table 2, Table 3 appear below.

**Table 2:** 1,2,4-Oxadiazol-3-yl benzoic acid derivatives in RAR α, β and γ transactivation assays.^a^

**Figure:**
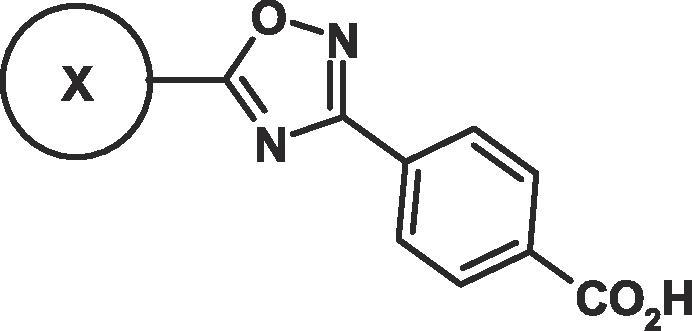

Compd	X	β EC_50_ nM^a^	α EC_50_ nM^a^	γ EC_50_ nM^a^	Fold Selectivity for β over α^b^	Fold Selectivity for β over γ^b^	cLog P^d^
**ATRA**	–	**1.9**	1.2	0.9	**0.6**	0.5	
**5**		**1.5**	18^c^	28	**12**	19	5.1
**8**		**4200**	18	17	**0.0043**	0.0041	7.2
**9**		**1.4**	4	3	**2.8**	2.1	7.2
**10**		**1.9**	26	11	**13**	5.6	5.3
**11**		**2.5**	19	5.3	**7.6**	2	5.3
**12**		**3.4**	30	6.3	**9**	2	5.8
**13**		**11**	114	83	**10**	7.5	4.1

a–d See Table 1.

**Table 3:** Derivatives of 4-(5-(4,7-dimethylbenzofuran-2-yl)-1,2,4-oxadiazol-3-yl)benzoic acid in the RAR α, β and γ transactivation assays.^a^

**Figure:**
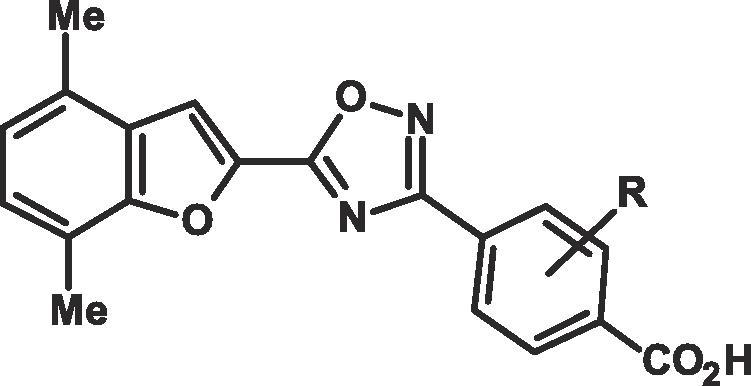

Compd	R	β EC_50_ nM^a^	α EC_50_ nM^a^	γ EC_50_ nM^a^	Fold Selectivity for β over α^b^	Fold Selectivity for β over γ^b^	cLog P^d^
**10**	**H**	**1.9**	26	11	**13**	5.6	5.3
**14**	**2-F**	**2.2**	16	8.4	**7.3**	3.8	5.1
**15**	**2-Me**	**14**	89	25	**6.4**	1.8	5.5
**16**	**3-F**	**11**	61	3.7	**5.5**	0.33	5.5
**17**	**3-Me**	**47**	600	14	**13**	0.3	5.5

a,b,d See Table 1.

The authors would like to apologise for any inconvenience caused.

