# Supplementary material for: Discovery and lead optimisation of a potent, selective and orally bioavailable RARβ agonist for the potential treatment of nerve injury
Source: Bioorg Med Chem Lett. 2019 Apr 15;29(8):995–1000. doi: 10.1016/j.bmcl.2019.02.011 (PMC6419571; doi:10.1016/j.bmcl.2019.02.011)
Supplement: Supplementary Data 1 [file mmc1.doc]

**Supplementary Data**

**Discovery and lead optimisation of a potent, selective and orally bioavailable RARβ agonist for the potential treatment of nerve injury.**

Maria B. Goncalves,**^a^** Earl Clarke,**^a^** Christopher Jarvis,**^a^** S. Barret Kalindjian,**^a^** Thomas Pitcher**^a^**, John Grist**^a^**, Carl Hobbs**^a^**, Thomas Carlstedt**^a^**, Julian Jack**^a^,** Jane T. Brown,**^b^** Mark Mills,**^b^** Peter Mumford,**^b^** Alan D. Borthwick,^c^* and Jonathan P. T. Corcoran**^a^***

*^a^Neuroscience Drug Discovery Unit, Wolfson Centre for Age-Related Diseases, Guy’s Campus, King’s College, London SE1 1UL, UK.*

*^c^DrugMolDesign, 15 Temple Grove, London NW11 7UA, UK.*

*^b^Sygnature Discovery Limited, Biocity, Pennyfoot Street, Nottingham NG1 1GF, UK.*

* *Corresponding authors.*

*E-mail address: alan.d.borthwick@drugmoldesign.com (A.D. Borthwick).*

**Experimental protocols.**

Analytical LCMS was performed using an Agilent 1200 HPLC and mass spectrometer system with a Scalar 5 mm C18 4.6 x 50 mm column and peaks detected by positive or negative ion electrospray ionization and a UV detector at 254 nm. All tested compounds were found to be of >95% purity using analytical LCMS. ^1^H and ^13^C NMR spectra were recorded using a Varian Unity INOVA 400 MHz NMR spectrometer with a 5mm ID probe, using tetramethylsilane as a reference in DMSO-d_6_ as a solvent.

All compounds in **Tables 1-3** were synthesized and characterised as described previously (WO 2016097004). Here, the synthesis and characterisation of our lead oxadiazole **10** is shown as an example.

**1. Chemical synthesis**

**Methyl 4-(5-(4,7-dimethylbenzofuran-2-yl)-1,2,4-oxadiazol-3-yl)benzoate** (**20**)

A solution of T3P in ethyl acetate (50%) (23.2 mL, 39.4 mmol) was added dropwise to a mixture of 4,7-dimethylbenzofuran-2-carboxylic acid (**18**) (3.0 g, 16 mmol), methyl 4-(*N*'-hydroxycarbamimidoyl)benzoate (**19**) (3.1 g, 16 mmol) and triethylamine (11 mL, 79 mmol) in anhydrous dimethylformamide (25 mL), stirring at 0°C. The mixture was stirred at 0°C for 10 min then warmed to 90°C and stirred for 18 h. The reaction mixture was cooled to room temperature and poured into iced water (150 mL). The solid was collected, washed with cold ethyl acetate and dried under suction. The material was purified by trituration with methanol and dried *in vacuo* to afford the title compound (**20**) (3.6 g, 65% yield) as a pink solid: m/z 349 [M+H]^+^ (ES^+^). ^1^H NMR (400 MHz, DMSO‑*d_6_*) δ: 8.29-8.26 (3H, m), 8.19 (2H, d), 7.29 (1H, d), 7.13 (1H, d), 3.92 (3H, s), 2.56 (3H, s), 2.54 (3H, s).

**4-(5-(4,7-Dimethylbenzofuran-2-yl)-1,2,4-oxadiazol-3-yl)benzoic acid** (**10**)

A suspension of methyl 4-(5-(4,7-dimethylbenzofuran-2-yl)-1,2,4-oxadiazol-3-yl)benzoate (**20**) (100 mg, 0.287 mmol) in tetrahydrofuran (1 mL) was treated with lithium hydroxide

(2 M, aq., 720 µL, 1.4 mmol) and the mixture was stirred at 40°C for 20 h. The reaction mixture was cooled to room temperature, then acidified by the dropwise addition of 1 M hydrochloric acid. The resulting solid was collected by filtration, then dissolved in methanol and evaporated to dryness to afford the title compound (**10**) as a white solid (95 mg, 99% yield). Recrystallisation from acetone gave white needles mp 270°C. ^1^H NMR (400 MHz, DMSO‑*d*_6_) δ: 8.15 (2H, d), 8.10 (2H, d), 8.00 (1H, s), 7.20 (1H, d), 7.04 (1H, d), 2.51 (3H, s), 2.50 (3H, s). ^13^C NMR (400 MHz, DMSO *d*_6_) δ 168.5, 168.2, 166.9, 154.9, 139.9, 134.1, 130.7, 130.4, 129.9, 129.1, 127.7, 126.8, 124.8, 119.2, 113.2, 18.1, 14.7. m/z 335 [M+H]^+^ (ES^+^), 333 [M-H]^-^ (ES^-^). HRMS requires C_19_H_15_N_2_O_4_ (MH+) 335.1028, found 335.1025 (error 0.3 ppm). Anal. Found: C, 68.6; H, 4.3; N, 8.5 C_19_H_14_N_2_O_4_ (334.3) requires C, 68.3; H, 4.2; N, 8.4

**2. Transactivation assays.**

Compounds were tested in transactivation assays at the RAR α, β and γ receptors. Full dose-response curves were generated for each agonist and the potency of each compound was expressed as its EC_50_ together with that of reference ATRA EC_50_ value generated on each 96 well plate.

**Transactivation assays for RAR alpha, beta and gamma receptors:**

Transcriptional transactivation assays have been performed with *gal4* fusion receptor constructs, created using each of the mouse RAR ligand-binding domains, co-transfected with the pFR-luc (Stratagene) reporter construct in COS-7 cells. Thus, transfected cells will constitutively express the gal4-RAR fusion protein which in turn may be transactivated by ATRA to induce the expression of the *luciferase* that is driven by a gal4UAS.

Briefly, on day one, 96 well plates were seeded with 8000 cells per well then left to recover overnight. On day two, the cells were co-transfected with 100ng of reporter plasmid and 10ng of the appropriate receptor plasmid per well using lipofectamine (Invitrogen). On day three, the lipofectamine containing media was replaced by a DMEM without phenol red, followed by the addition of novel compounds dissolved in 1µl of DMSO to each well’s 100µl total volume. Finally, on day four, the cells were lysed and their luciferase substrate was provided by the BrightGlo reagent (Promega), the plates were then read on the MicroBeta TriLux (Perkin Elmer).

In each experiment an 8 point dose response curve of ATRA was run in duplicate, and dose response curve of test compounds tested were also generated in duplicate. EC_50_ data both for test compounds and ATRA was generated by fitting dose-reponse curves using GraphPad Prism^TM^. Data for test compounds are quoted as EC_50_ values. Where replicate data has been generated the data are quoted as the mean EC_50_ from the separate experiments.
